# Induction of anti-tumor immunity by trifunctional antibodies in patients with peritoneal carcinomatosis

**DOI:** 10.1186/1756-9966-28-18

**Published:** 2009-02-14

**Authors:** Michael A Ströhlein, Robert Siegel, Michael Jäger, Horst Lindhofer, Karl-Walter Jauch, Markus M Heiss

**Affiliations:** 1 Department of Abdominal, Vascular and Transplant Surgery, Campus Cologne-Merheim, Witten/Herdecke University, Ostmerheimer Str. 200, 51109 Cologne, Germany; 2 Department of Surgery, Klinikum Grosshadern, Ludwig-Maximilians-University of Munich, Marchionistr. 15, 81377 Munich, Germany; 3 TRION Research, Frankfurter Ring 193A, 80523 Munich, Germany

## Abstract

Peritoneal carcinomatosis (PC) from epithelial tumors is a fatal diagnosis without efficient treatment. Trifunctional antibodies (trAb) are novel therapeutic approaches leading to a concerted anti-tumor activity resulting in tumor cell destruction. In addition, preclinical data in mouse tumor models demonstrated the induction of long lasting tumor immunity after treatment with trAb. We describe the induction of anti-tumor specific T-lymphocytes after intraperitoneal administration of trAb in patients with PC.

9 patients with progressive PC from gastric (n = 6) and ovarian cancer (n = 2), and cancer of unknown primary (n = 1) received 3 escalating doses of trAb after surgery and/or ineffective chemotherapy. The trAb EpCAM × CD3 (10, 20, 40 μg) or HER2/neu × CD3 (10, 40, 80 μg) were applicated by intraperitoneal infusion. Four weeks after the last trAb application, all patients were restimulated by subdermal injection of trAb + autologous PBMC + irradiated autologous tumor cells. Immunological reactivity was tested by analyzing PBMC for specific tumor reactive CD4+/CD8+ T lymphocytes using an IFN-γ secretion assay.

In 5 of 9 patients, tumor reactive CD4+/CD8+ T-lymphocytes increased significantly, indicating specific anti-tumor immunity. A clinical response (stable disease, partial regression) has been observed in 5 of 9 patients, with a mean time to progression of 3.6 months. Follow-up showed a mean survival of 11.8 months (median 8.0 months) after trAb therapy.

TrAb are able to induce anti-tumor immunity after intraperitoneal application and restimulation. The induction of long-lasting anti-tumor immunity may provide an additional benefit of the intraperitoneal therapy with trAb and should be further elevated in larger clinical trials.

## Background

Peritoneal carcinomatosis (PC) is a common disseminated type of gastric and ovarian cancer. It is associated with a poor prognosis with a median survival of only few months [1,2]. PC is accompanied by obsessing symptoms like malignant ascites and ileus due to abdominal obstruction, which is treated by paracentesis or palliative surgery. No efficient standard treatment to prevent or eradicate peritoneal spread is available so far. Conventional intravenous (i.v.) chemotherapy was generally not effective [3,4]. Various experimental and multimodal concepts have been evaluated including peritonectomy procedures[5,6], hyperthermic intraperitoneal (i.p.) chemotherapy [7,8] or immediate postoperative i.p. chemotherapy [9,10]. All these concepts indicated that local treatment procedures might represent the best option for treatment of PC.

New therapeutic concepts employ trifunctional antibodies (trAb) that recruit and activate different types of immune effector cells at the tumor site. TrAb are artificially engineered immunoglobulins with two different Fab-binding sites and an intact Fc-region [11] and represent a novel antibody concept [12]. They effectively enhance the anti-tumor activity not only by induction of T-cells by CD3-binding, but also by simultaneous activation of accessory cells [13,14]. Responsible for this feature is a potent isotype combination (mouse IgG2a and rat IgG2b), which binds and activates FcγRI and RIII positive cells (e.g. dendritic cells, macrophages, granulocytes and NK-cells). The tri-cell complex of T-lymphocytes, tumor cells and accessory cells induces efficient tumor cell killing, which results from an activating "crosstalk" via cytokines (like e.g. IL-2, IL-12 and TNF-α) and costimulatory molecules between different immune cell types [13]. Therefore, trAbs are able to activate cell-mediated cytotoxicity leading to MHC-unrestricted but specific killing of targeted tumor cells without requirement for any pre-activation or co-stimulation. Moreover, involvement and activation of Fcγ RI/III positive professional antigen presenting cells results in phagocytosis of tumor cells and subsequent induction of anti-tumor immunity by tumor antigen processing and presentation [14,15]. This phenomenon was supposed to result in polyclonal humoral and cellular immune responses, including T-cell responses even against unknown, tumor-associated peptides. This hypothesis was confirmed in a syngeneic mouse tumor model, where i.p. treatment with trAb demonstrated striking anti-tumor effects including tumor destruction and long term immunity, which where independent of the primary tumor binding site of the applicated trAb [15]. The trAb catumaxomab has dual specifity for epithelial cell adhesion molecule (EpCAM) and CD3; ertumaxomab targets epidermal growth factor family member (HER2/neu) and CD3. EpCAM is frequently expressed in different gastrointestinal malignancies like colon and stomach and in lung and ovarian cancer [16,17], HER2/neu is overexpressed in breast cancer [18]. EpCAM and HER2/neu are both a prognostic marker and a target antigen [19,20].

In a previous study, we could demonstrate in vivo cytotoxicity mediated by trAb catumaxomab in patients with malignant ascites [21]. A multicenter phase I/II study showed that an i.p. immunotherapy with catumaxomab prevented accumulation of ascites and eliminated tumor cells with an acceptable safety profile [22].

In this prospective pilot study, we investigated the induction of anti-tumor specific T-lymphocytes after i.p. administration and restimulation with trAb in patients with PC.

## Patients and methods

### Objectives and study approval

This study was designed as a sequential dose-escalating, feasibility study for compassionate use of trAb in the induction of tumor immunity.

The study was carried out according to the principles of the Declaration of Helsinki and good clinical practice guidelines. It was approved by the Ethics committee of the Ludwig-Maximilians-University, Munich, Germany. Informed consent was obtained from all patients prior to treatment.

### Patients

Patients enrolled in this study had histologically confirmed diagnosis of PC.

Inclusion criteria were Karnofsky performance status ≥ 60%, white blood cell count > 2000/mm^3 ^and a relative T-cell count > 10%.

Exclusion criteria included prior immunotherapy, significant heart disease or arrhythmia, known allergic reactions or autoimmune disease, significant liver, kidney, pulmonary or haematological disease, acute or chronical infection and paracentesis of malignant ascites > 1000 ml within 30 days before treatment.

Patients were included independent of any prior conventional therapy, i.e. chemotherapy, radiation or tumor surgery. An interval of more than 30 days between any chemotherapy and the start of the trAb therapy was required. A recovery interval of at least 7 days after abdominal surgery with laparotomy was mandatory.

All patients had a surgical procedure (explorative laparotomy or laparoscopy, resection of intra-abdominal metastases), where isolation of autologous tumor samples was possible.

### Isolation and storage of autologous tumor cells

Autologous tumor samples were taken during surgery (explorative laparotomy or laparoscopy, resection of intra-abdominal metastasis). The surgical procedure was independent from study inclusion. Patients were only included if more than 5 × 10^6 ^autologous tumor cells were successfully isolated, and if EpCAM antigen or HER2/neu antigen was found on > 10% of all viable cells from autologous tumor cell preparations.

Analysis of autologous tumor cells was performed by immunohistochemical APAAP staining [23] using the antibodies HO3 (anti-EpCAM; mouse IgG2a, TRION Pharma) or C215 (anti EpCAM; mouse IgG2a; kindly provided by M. Dohlsten, Pharmacia, Uppsala, Sweden) for EpCAM or 2502A (anti Her2/neu; mouse IgG2a; Trion Pharma, Munich, Germany) for HER2/neu.

After surgical resection autologous tumor probes were dissected into 2–3 mm^3 ^pieces which were then immersed in RPMI 1640 medium (containing 0.05% Collagenase type 4, 0.02% DNAse type 1, Penstrep, Gentamycin and Amphotericin B; all reagents from Invitrogen, Carlsbad, California). This mixture was incubated overnight at 37°C and filtered through a flexible grid to exclude undigested tissue fragments. The resulting tumor cell suspension was washed twice with HBSS and resuspended in RPMI 1640 medium (containing Glutamin and Pyruvate; Invitrogen,) and 10% autologous serum at a concentration of 5 to 10 × 10^6 ^tumor cells. After cooling down to 4°C, 10% DMSO (PAN Biotech, Aidenbach, Germany) was added. Then, standardized freezing by 1°C per minute was performed using a computer controlled freezing device (Air Liquide, Duesseldorf, Germany). Frozen autologous tumor cells were stored at -196°C.

### TrAb

TrAbs catumaxomab (anti-EpCAM × anti-CD3, removab^®^) and ertumaxomab anti-Her2/neu × anti-CD3 (rexomun^®^) were produced under GMP conditions as previously described [11] and provided by Trion Pharma, Munich, Germany.

### Treatment

Patients received an i.p. catheter or port system for trAb application. In order to achieve a standard minimal intraperitoneal volume of distribution, 1000 ml of balanced electrolyte solution were infused i.p. before every trAb application. TrAb were administered via the i.p. catheter as a continuous infusion over 6 hours. In order to prevent clinical symptoms within the known antibody treatment-associated „cytokine release syndrome“ [24], pre-medication consisted of paracetamole supp. 1000 mg and dimetindene i.v. 50 mg, applicated 30 min before trAb-infusion.

Patients received three escalating doses of trAb (10, 20, 40 μg of EpCAM × CD3; or 10, 40, 80 μg of HER2/neu × CD3). Between two trAb applications, an interval of 2 to 3 days was inserted. The first application consisted of 10 μg of trAb. Criteria for the next trAb application were well-being of the patient, leucocyte counts < 13 G/L and body temperature < 37.5° for at least 12 hours. Dose reduction was dependent on the individual reaction to the prior dose, i.e. inflammatory reactions and side effects.

### Antigen boost – Vaccination

Restimulation was performed by exposition of the patients to autologous tumor cells and trAb 30 days after the last i.p. infusion. Cryo-conservated autologous tumor cells were rapidly thawed in a 37°C water bath and washed in balanced electrolyte solution, followed by a 100 gray irradiation. 10 × 10^6 ^autologous PBMC were isolated by a standard Ficoll-Hypaplaque (PAN Biotech, Aidenbach, Germany) density centrifugation technique. PBMC and 1 × 10^6 ^autologous tumor cells were resuspended in a balanced electrolyte solution and incubated in vitro for 30 minutes together with 3 μg of trAb anti-EpCAM × anti-CD3 or anti-HER2/neu × anti-CD3 depending on the individual antigen expression of autologous tumor cells. The vaccination was performed by an intradermal injection at two sites on both limbs.

### Evaluation of immunological reactivity

In order to compare immune reactivity by CD4+/CD8+ T-lymphocytes against autologous tumor cells, venous blood samples were taken before commencing therapy and 7 to 10 days after boost vaccination. 1 × 10^7 ^PBMC were isolated by Ficoll-Hypaplaque density centrifugation. PBMC were stimulated in 24 well plates with autologous tumor cells only. After an 8 hour stimulation period, the Miltenyi Biotec^® ^IFN-γ Secretion Assay was performed according to the manufacturers manual (Miltenyi Biotech, Bergisch Gladbach, Germany), using anti-human CD4 and anti-human CD8 antibodies (Coulter Immunotech, Krefeld, Germany). FACS analysis was performed with a FACSCalibur Flow cytometer (Becton Dickinson, Heidelberg, Germany) using CellQuest Pro and WinMDI software. Unstimulated PBMC and PBMC after incubation with allogeneic EpCAM+ HT-29 (ATCC Nr. CCL-244) or HER2/neu+ SK-BR-3 carcinoma cells (ATCC Nr. HTB-30) were used as negative controls.

### Clinical patient evaluation/toxicity and safety evaluation

Careful patient monitoring was applied throughout the study. Clinical evaluation, including medical history and general physical exam, was performed at baseline and defined days during treatment (day of trAb infusion and the following day; day of restimulation and the following day). Patients were monitored for adverse events according to the National Cancer Institute common toxicity criteria during each visit. Standard laboratory parameters and vital signs were tested before and after treatment Laboratory testing included complete blood count, electrolytes, creatinine, bilirubin, transaminases, and tumor marker (CA19-9 for gastric carcinoma, CA125 for ovarian carcinoma, CEA and CA125 for CUP). In addition, patients during trAb therapy were daily monitored for systemic cytokine responses. Blood samples were taken before, 24 hours and 48 h after every trAb application. Serum levels of IL-6, TNF-α, and sIL-2R were measured by ELISA (Biosource, Fleurs, Belgium). Immune reaction to mouse IgG was assessed by ELISA measurement of human anti-mouse antibody reaction (HAMA) before and 4 weeks after therapy.

Response was evaluated by computed tomography two to three months after trAb treatment and every two to three months until tumor progression.

### Statistical analysis

Analysis of cytokine levels was performed using the Wilcoxon signed rank test. Correlation analysis was done by the chi-square contingency analysis. All tests were calculated by SAS statistical software using a Windows XP computer system.

## Results

### Patients' characteristics

Nine patients were treated between February 2005 and December 2007. Prior to study treatment, 6 patients underwent surgical resection with curative intent, 8 patients received chemotherapy. Four patients presented with synchronous PC, whereas five developed PC after surgery and chemotherapy. One patient was diagnosed with PC of carcinoma of unknown primary (CUP) during elective laparoscopic cholecystectomy. The patients' demographic and primary treatment parameters are listed in Table 1.

**Table 1:** Patients' characteristics

**Pat.**	**Age**	**Sex**	**Tumor entity**	**TNM stage primary**	**Surgical therapy primary tumor**	**Chemotherapy before trAb**	**Radiation before trAb**	**EpCAM expression**	**HER2/neu expression**
A	31	f	Gastric	pT4pN3M0	Gastrectomy	+	+	+	+
B	64	f	Ovarian	pT3pN0M0	Adnexectomy, resect. of liver met.	+	-	+	-
C	50	f	CUP	-	-	-	-	+	-
D	62	f	Ovarian	pT3pN2M0	Adnexectomy	+	-	-	+
E	59	m	Gastric	pT4pN2M1	Gastrectomy	+	-	+	+
F	35	f	Gastric	pT3pN0M0	Gastrectomy	+	-	+	-
G	38	f	Gastric	T3N+M1	-	+	-	+	-
H	51	m	Gastric	T3N+M1	-	+	-	+	-
I	62	m	Gastric	pT3pN2M1	Gastrectomy	+	-	+	-

EpCAM or HER2/neu expression of autologous tumor cells was done by APAAP immunohistochemical staining. EpCAM+ or HER2/neu+: > 10% stained cells in autologous tumor cell preparations; CUP = carcinoma of unknown primary.

### Application of trAb and monitoring

All nine patients received i.p. trAb applications. No dose escalation for the third application was performed in patient A because of side effects. In patient C, reduced starting dose of 5 μg was in respect of a body weight of 43 kg only; Patient F refused the third application of trAb. For detailed therapy of each patient, please see Table 2 and Table 3.

**Table 2:** I.p. application of trAb anti-EpCAM and side effects

**Pat.**	**TrAb anti-EpCAM therapy (μg i.p./day)**						**Cumulative dose**	**Side effects**
	**μg**	**day**	**μg**	**day**	**μg**	**day**	**(μg)**	
A	10	1	20	5	20	9	50	Elev. of AP (3), γ-GT (4); fever (3); abdominal pain (3); vomiting (3)

B	10	1	20	6	40	9	70	Elev. of AP (2), bilirubin (2), γ-GT (3), GOT (3), GPT (3); fever (3); abd. pain (3); vomiting (2); allergic exanthema (2)

C	5	1	20	3	40	7	65	Fever (2)

F	10	1	20	5	-		30	Elev. of AP (2), PTT (2), GPT (3); fever (1); abdominal pain (3); vomiting (2)

G	10	1	20	5	40	10	70	Elev. of AP (1), bilirubin (2), γ-GT (3), GPT (3); fever (1); abdominal pain (3)

H	10	1	20	7	40	13	70	Elev. of AP (1), bilirubin (2), gGT (3), creatinine (2); fever (1); abdominal pain (3)

I	10	1	20	8	40	12	70	Elev. of AP (1); fever (2); vomiting (3)

**Table 3:** I.p. application of trAb anti-Her2/neu and side effects

**Pat.**	**TrAb anti Her2/neu therapy (μg i.p./day)**						**Cumulative dose**	**Side effects**
	**μg**	**Day**	**μg**	**Day**	**μg**	**day**	**(μg)**	
D	10	1	40	4	80	8	130	Fever (1)

E	10	1	40	6	80	8	130	Fever (1); abdominal pain (2)

Individual schedule of trAb therapy and side effects according to the National Cancer Institute (NCI) common toxicity criteria.

TrAb treatment was accompanied by transient fever (up to 40.4°C) after 9 applications. The fever developed six to ten hours after trAb infusion and disappeared within the next day. Metamizole (1000 mg) was given in these cases. Six patients complained about abdominal pain; four patients had vomiting and required treatment with Dimenhydrinate. No patient required ICU admittance.

Elevated liver enzymes, elevated levels of γ-glutamyl transferase and alkaline phosphatase were observed after trAb application. These laboratory changes disappeared spontaneously within the treatment intervals.

**Figure 1:**
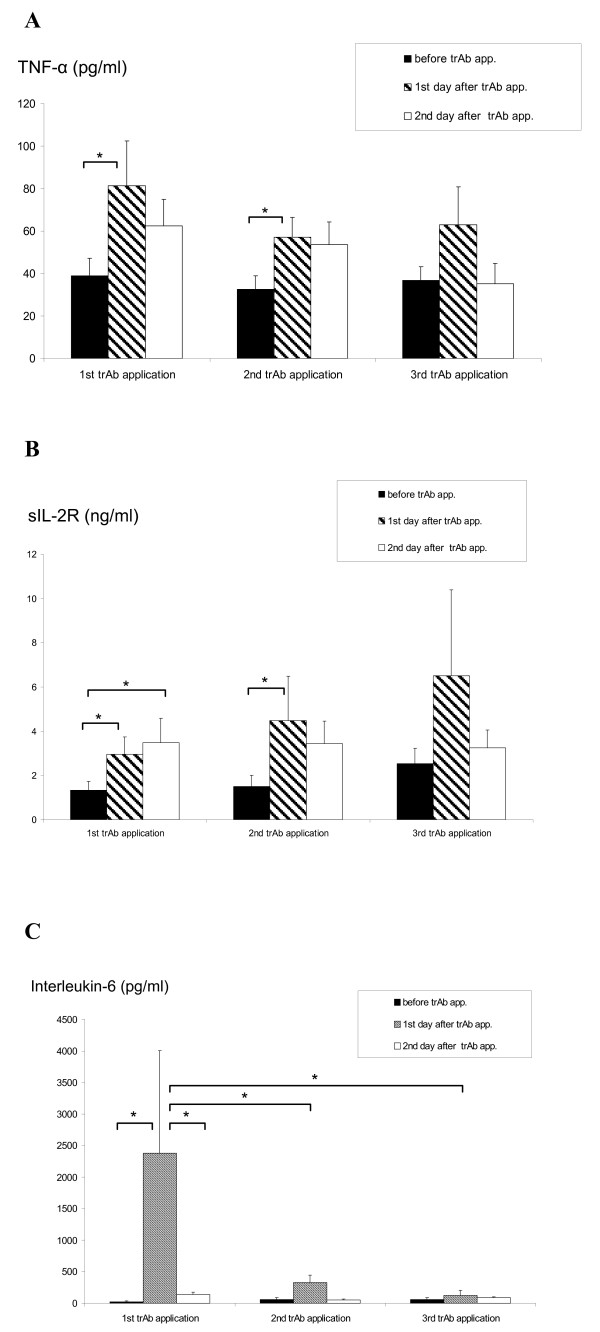
**Serum levels (mean, +/- SEM) of TNF-α (A), soluble IL-2R (B), and IL-6 (C) immediately before the first, second and third trAb application, and corresponding serum levels on day one and two dafter trAb therapy**. Serum levels were measured by ELISA (Biosource, Fleurs, Belgium). * p < 0.05.

TrAb treatment was followed by an elevation of serum levels of IL-6, TNF-α, and soluble IL-2 receptor one day after treatment. The slight decrease on the second day after every trAb application was statistically not significant (Figure 1A, 1B). The inflammatory cytokine IL-6 showed a substantial increase after the first trAb infusion only; despite trAb dose escalation there were only moderate increases after the following two applications (Figure 1C). TNF-α was increased after every trAb application, followed by a slight decrease on day 2. The soluble IL2-receptor (sIL2R) serum level, which indicates T-cell activation, analogously increased after each trAb application. Comparing the sIL2R level on day 1 after trAb application, the maximum sIL2R level was found after the third trAb application, indicating an ongoing and increasing cellular immune activation during trAb therapy.

HAMA was measured after trAb therapy in 7 of 9 patients. In all these patients, HAMA was significantly increased (above the threshold of 40 ng/ml), representing an immunological reaction (Table 4).

**Table 4:** Restimulation and response

**Patient**	**Increase of IFN-γ secreting T-lymphocytes**	**HAMA (ng/ml)**	**Chemotherapy after trAb therapy**	**Survival after trAb therapy (months)**
A	+	801	-	1
B	+	230	+	21
C	-	30512	+	31
D	-	n.d.	-	4
E	+	7870	+	7
F	-	50730	+	12
G	+	2540	+	15
H	-	400	+	8
I	+	n.d.	-	7

Increase of IFN-γ secreting T-lymphocytes compared to baseline values before therapy. HAMA = human anti-mouse antibody reaction (values measured 4 weeks after trAb therapy).

### Immunological anti-tumor reactivity

**Figure 2:**
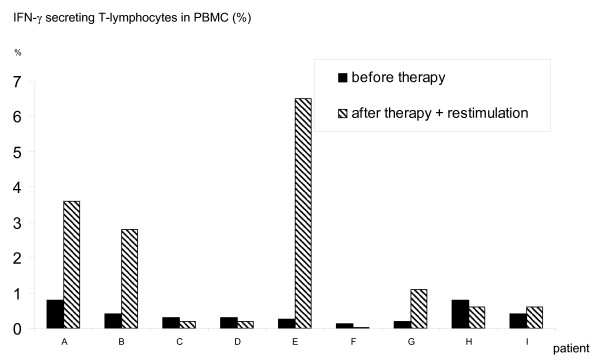
**Individual percentage values presenting the relative proportion of IFN-γ secreting T-lymphocytes in 10 × 10^6 ^PBMC after stimulation with 5 × 10^5 ^autologous tumor cells before and 3–4 weeks after trAb therapy using the Miltenyi IFN-γ secretion assay**.

**Figure 3:**
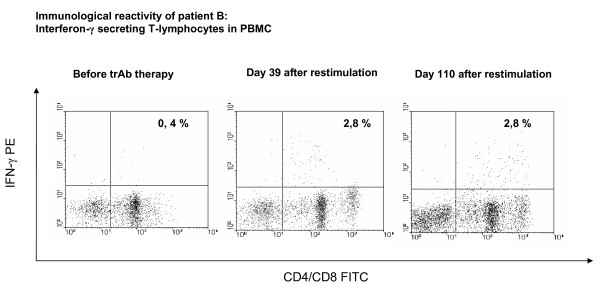
**Analysis of tumor reactive IFN-γ secreting CD4+/CD8+ T lymphocytes before trAb therapy and on day 39 and 110 after boost stimulation in patient B using the Miltenyi IFN-γ secretion assay**.

All patients were restimulated 4 weeks after i.p.-application of trAb. Patients revealed a base value of 0.4% (mean) CD4+/CD8+ IFN-γ secreting T-lymphocytes in PBMC before trAb-treatment. Five of nine patients showed an increase of IFN-γ secreting T-lymphocytes, reflecting autologous anti-tumor reactivity (Figure 2). In these 5 patients, the number of tumor reactive T-lymphocytes increased from baseline value of 0.4% to 2.9% (mean) after trAb therapy and restimulation. All control experiments with unstimulated PBMC or PBMC incubated with allogeneic tumor cells showed no increase compared to the corresponding baseline values. In patient B, the IFN-γ secretion assay was performed twice after intradermal restimulation (Figure 3). Here, IFN-γ secreting T-lymphocytes increased from 0.4% before therapy to 2.8% after restimulation, followed by a value of 2.8% on day 110 after stimulation, indicating long-term immunity. This patient also had a substantial decrease of tumor markers (CA 125 decreased from 57.8 U/ml to 29.7 U/ml).

### Clinical outcome

Study patients did not receive any other tumor specific therapies during i.p. trAb infusion or antigen restimulation. According to RECIST criteria, 5 of 9 patients (Patients B, C, F, G, H) showed a clinically stable disease or partial tumor regression with a mean time to progression of 3.6 months (range 1 to 6 months) without any further tumor specific treatment.

After trAb therapy and restimulation, overall survival was 8.0 months (median; range 1 to 31 months). 6 patients received chemotherapy after trAb immunotherapy. In none of the patients accumulation of malignant ascites was observed after trAb therapy.

## Discussion

The results of this pilot study on the i.p. application and restimulation by trAb in patients with PC provide strong evidence for the induction of specific immune reactions against autologous tumor cells by T-lymphocytes upon trifunctional antibody treatment. Further more the study confirmed the safety and feasibility data of i.p. application of trAb in patients without accumulation of ascites.

TrAb application was accompanied with "immunological" side effects like fever, elevation of inflammatory markers and allergic skin reactions. Further symptoms like abdominal pain and nausea could be attributed to the disturbance of the peritoneum by trAb mediated local inflammation. Transient elevation of liver enzymes, γ-glutamyl transferase and alkaline phosphatase were observed after application of the anti-EpCAM × anti-CD3 trAb, but were not correlated to clinical symptoms. As the epithelium of the biliary system typically expresses the EpCAM-antigen [25], this side effect could be presumably attributed to a transient trAb-induced cholangitis. In summary, all these side effects are very well in concordance with the recently published results of our studies investigating the trAb therapy in malignant ascites [21,22].

Major aim of this study was to investigate the induction of T-cell mediated immune responses to autologous tumor cells by intraperitoneal treatment and restimulation, as induction of long-term immunity by trifunctional antibodies was successfully demonstrated in an animal model [15]. In five out of nine patients, specific tumor reactive CD4/CD8 + T lymphocytes were found in PBMC by the IFN-γ secretion assay, demonstrating that i.p. trAb therapy is able to induce a verifiable increase of autologous tumor reactive T lymphocytes. Additionally, sIL-2 levels also indicated T-cell activation. Therefore we conclude that formation of the so called tri-cell-complex of T-lymphocytes, tumor cells and accessory cells by trifunctional antibodies may result in induction of T-cell mediated anti-tumor reactivity.

Regarding the structural binding sites of trifunctional antibodies, one of the unique capacities of trAb is to bind and activate CD3+ lymphocytes and CD64+ accessory cells simultaneously. Several previous studies were performed using anti-CD3 × anti-tumor bispecific antibodies (bsAb) in non Hodgkin's lymphoma and solid tumors like ovarian and renal cell cancer [26-28]. But these studies but were mainly limited by the lack of costimulary signals after primary activation. In addition, CD64 was described as an attractive target molecule for bsAb based immunotherapy of cancer [29]; anti-EpCAM × anti-CD64 bsAb were characterized to mediate strong cytotoxicity in vitro after GCSF and IFN-γ pre-stimulation of PBMC [30]. Moreover, two studies using the bsAb MDX-H210 (anti-HER2/neu × anti CD64) demonstrated clinical feasibility but limited clinical efficacy in several patients with a dosage 15 mg/m2 after GCSF or GMCSF stimulation [31,32]. In this context, it should be highlighted that trAb significantly differ from all described bsAb constructs. TrAb consist of the two potent subclasses mouse IgG2a and rat IgG2b, which determine the unique effector functions. In contrast to similar T-cell redirecting bsAb, this mechanism does not depend on the addition of exogenous cytokines or co-stimulation to provide full anti-tumor activity [14] as the formation of a postulated tri-cell complex between tumor cell, T-cell and accessory cell represents a fully self-supporting system for efficient immune cell activation [13].

PC is generally seen as terminal tumor stage with rapid progression. Regarding the natural history of PC, where exponential tumor growth is expected, the observed clinical course with stable disease or partial tumor regression in five patients and the observed mean survival of 11.8 months (median 8.0 months) after trAb therapy is remarkable. None of the nine patients developed accumulation of malignant ascites during therapy, which would have been expected in 20 to 30% of patients with PC. Although outcome is not the goal of this trial, compared to a mean survival of 6 months (median 3.1 months) from the landmark study by Sadeghi et al. in 370 patients with PC [1] our results are promising.

In summary, our results demonstrate that trAb are capable to induce specific tumor immunity against autologous tumor cells. In addition to the well documented ability of tumor cell destruction [21], especially this unique self-supporting efficacy of trAb may provide a new concept in the treatment of intraabdominal tumors. Ongoing studies in early stages of PC and in patients with high risk for development of peritoneal tumor disease will further evaluate the therapeutic impact of trAb.

## Competing interests

The study reported in the manuscript was partially funded by TRION Pharma, Munich, Germany. The authors certify that they have not entered into any agreement that could interfere with their access to the data on the research, nor upon their ability to analyze the data independently, to prepare manuscripts, and to publish them.

MMH, MAS, HL and MJ have declared a financial interest in TRION Pharma, Germany, whose product was studied in the work presented in this paper.

## Authors' contributions

MAS and RS drafted the manuscript and provided data interpretation. MAS, MJ and HL performed and analyzed the experiments. KWJ and MMH conceived of the study, and participated in its design and coordination. All authors read and approved the final manuscript.
